# The relationship between the home environment and child adiposity: a systematic review

**DOI:** 10.1186/s12966-020-01073-9

**Published:** 2021-01-06

**Authors:** Alice R. Kininmonth, Andrea D. Smith, Clare H. Llewellyn, Louise Dye, Clare L. Lawton, Alison Fildes

**Affiliations:** 1 School of Psychology, University of Leeds, Leeds, LS2 9JT UK; 2 Research Department of Behavioural Science and Health, University College London, London, UK

**Keywords:** Adiposity, Home environment, Childhood obesity

## Abstract

**Background:**

Extensive research has demonstrated the role of the Home Environment (HE) in shaping children’s energy balance behaviours. Less is known about direct relationships with bodyweight. This review examines associations between the social and physical aspects of three pre-defined Home Environment domains (food, physical activity and media) and adiposity measures in children ≤12 years.

**Methods:**

Six electronic databases (PubMed, Medline, EBSCO CINAHL, EMBASE, Web of Science, PsycInfo) were systematically searched up to October 2020. Studies reporting at least one physical and/or social aspect of the food, physical activity and/or media domains of the Home Environment in relation to child adiposity outcomes were included (*n* = 62).

**Results:**

Most studies examined one (*n* = 41) or two domains (*n* = 16). Only five studies assessed all three domains of the Home Environment. Most consistent relationships were observed for physical aspects of the home media environment; with greater availability of electronic devices associated with higher child adiposity (21/29 studies). Findings were less consistent for the smaller number of studies examining physical aspects of the home food or physical activity environments. 8/15 studies examining physical food environments reported null associations with adiposity. Findings were similarly mixed for physical activity environments; with 4/7 reporting null associations, 2/7 reporting negative associations and 1/7 reporting positive associations between access to physical activity equipment/garden space and adiposity. Fewer studies assessed social aspects (e.g. caregiver modelling or limit setting) of the Home Environment in relation to child adiposity and findings were again mixed; 9/16 media environment, 7/11 food environment and 9/13 physical activity environment studies reported null associations with child adiposity outcomes.

**Conclusions:**

The home media environment was most consistently associated with adiposity in childhood. Findings were less consistent for the home food and physical activity environments. Greater agreement on definitions and the measurement of the obesogenic home environment is required in order to clarify the strength and direction of relationships with child adiposity. Robust longitudinal research using comprehensive measures of the holistic home environment is needed to better identify which aspects contribute to excess weight gain in childhood.

**Trial registration:**

PROSPERO Systematic review registration number: CRD42018115139.

**Supplementary Information:**

The online version contains supplementary material available at 10.1186/s12966-020-01073-9.

## Introduction

Excess adiposity in childhood is a major public health issue, it is associated with a wide range of negative physical and psychological health outcomes [1, 2]. Socio-ecological models provide a useful framework for understanding the different factors contributing to childhood obesity risk [3], theorizing that children are shaped by the environments they interact with most often. The home environment and family context are where children spend a significant proportion of their time during key developmental years [4]. Around 70% of a child’s food (for children < 12 years old) is consumed at home and importantly, it is where children observe and learn from others’ behaviour [5–8]. Consequently, it is hypothesised that the home environment is a major factor in shaping children’s weight trajectories.

Numerous models have been developed to conceptualise how different aspects of the home environment may influence children’s growth and development [5, 9, 10]. Yet the multifaceted and complex nature of the home environment (HE) complicates attempts to characterise and measure its contribution to excess weight development in childhood. A variety of measures have been developed to capture different aspects of the obesogenic HE, such as the types and frequency of foods available in the home [11] or the availability of electronic devices in a child’s bedroom [12]. Relationships have been observed between these measures and children’s energy-balance behaviours, including dietary intake [13, 14], activity levels [15], and screen-based sedentary behaviours [16, 17]. However, the extent to which the HE is directly associated with child adiposity is less clear and no previous systematic reviews have examined this.

**Fig. 1:**
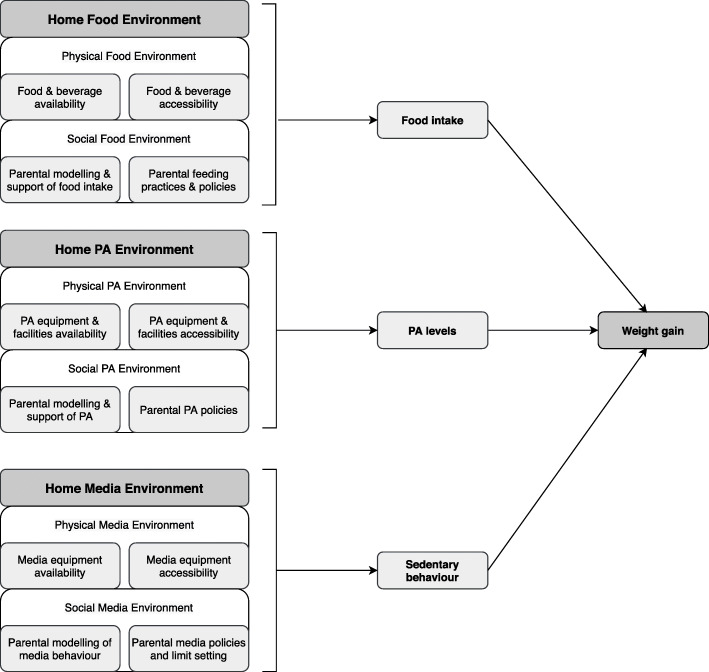
Conceptual model used to define the ‘obesogenic’ home environment [10]

For the purpose of this review, the obesogenic HE has been partitioned into three domains hypothesised to influence children’s food intake, activity levels and sedentary behaviours [9, 10, 14, 18]; *the food* (e.g. availability of sugar sweetened beverages [SSB]), *physical activity* (e.g. access to a garden) and *media-related* (e.g. caregiver rules around electronic devices) domains within the home. As shown in Fig. 1, each domain of the HE can be sub-divided into both physical aspects and social aspects that can either deter or promote health behaviours. These are all hypothesised to influence child energy balance and, ultimately, body weight.

Despite the wealth of literature in this field, previous reviews have largely focussed on only one domain of the HE, for example, the home food environment [19, 20], the home media environment [12], or the home physical activity environment [21]. Most of these reviews also explored relationships between the HE and behavioural outcomes such as children’s diet, activity and/or sedentary behaviour, meaning direct associations with adiposity have not been explored by previous reviews. Only one review from a decade ago included all three HE domains, but it primarily explored the psychometric properties of existing measures, rather than evaluating relationships with child weight [18]. No previous review has synthesised the evidence to investigate relationships between the obesogenic HE in its entirety, and child adiposity outcomes.

It is important to understand how the different aspects of the home environment relate to excess weight gain in childhood in order to inform effective targeted child obesity prevention and intervention strategies. Therefore, the purpose of this review is to examine associations between physical and social aspects of the food, activity and media domains of the HE with measures of adiposity in childhood (≤12 years).

## Methods

This review followed the PRISMA guidelines (Additional file 1 - PRISMA checklist) and was registered on PROSPERO (CRD42018115139).

### Eligibility criteria

Manuscripts were included if they reported on at least one of the three domains of the HE (food, activity and/or media). Each domain was required to be assessed in terms of either the physical (availability of, and access to; foods, media or PA equipment) and/or social aspects (caregiver modelling/support or caregiver policies and rules around energy balance behaviours). Studies were also required to provide a quantitative estimate of the association between the chosen HE domain(s) and a measure of adiposity (e.g. BMI z-score). Studies were eligible for inclusion if they were peer-reviewed original observational research studies and recruited from non-clinical, non-intervention populations. The population of interest was children aged ≤12 years. This age range was chosen to broadly capture the upper age of primary school children, which can vary both between and within countries (for example children in the U.K. and Australia typically start secondary school at age 11, whereas in Singapore and the Netherlands children tend to start secondary school at age 12). Additionally, the upper limit of 12 years was selected to focus this review on pre-teenage years, before children’s autonomy over their environment increases and they spend more time outside the home. Studies were excluded if they were not published in English and no translation was available (*n* = 41).

Family mealtimes were excluded from the definition of the social home food environment as a recently published review examined the relationship between family mealtimes and child weight [22]. This meta-analysis found higher family meal frequency was associated with better overall diet quality, greater consumption of nutrient dense foods and fewer energy-dense foods and lower child BMI.

### Literature search strategy

Six electronic databases were originally searched in November 2018, and this was then updated in October 2020: Medline (OVID from 1946 to Oct 2020), EBSCO CINAHL, EMBASE (OVID), Web of Science, PubMed, and PsycInfo (OVID). The search strategy (see Additional file 2) was informed by search terms from a relevant review [18]. Database searches were supplemented by reading the reference list of eligible studies and relevant reviews in the area [18].

### Identification of relevant studies and data extraction

Study eligibility was assessed independently by 2 reviewers; with 5% of title and abstracts and 10% of full texts screened in duplicate. There was 96% agreement between the reviewers, with any disagreement resolved via discussion. A standardised format for extraction was developed to ensure detailed data were obtained from each included study. Data extracted included key study and sample characteristics (e.g. study design, sample size, demographics), aspects of the HE examined (e.g. availability of physical activity equipment, caregiver modelling, etc.) and details of the child weight-related outcomes (e.g. measures used, population reference data, obesity cut-off criteria). The strength and direction of relationships between HE aspects and adiposity measures were also extracted.

### Assessment of study quality

Risk of bias was completed for each included study using an adapted version of the validated Newcastle Ottawa Scale (NOS) for cohort studies [23]. The tool was used to evaluate studies based on the research design, representativeness of the sample, appropriateness of the statistical analysis, recruitment strategy, measurement of exposure, and use of power calculation. A NOS score ≥ 7 was considered indicative of high study quality. The maximum score that could be awarded for study quality was 10. Full details are described in Additional file 3**.**

## Results

**Fig. 2:**
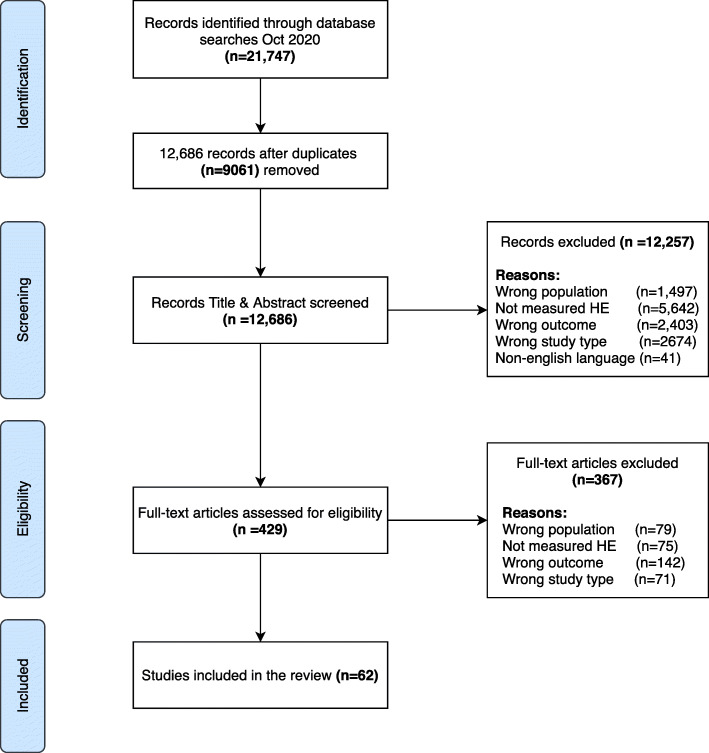
PRISMA Flow Diagram of the systematic review literature search

Overall, the search strategy identified 21,747 independent publications. Following title and abstract review, 12,257 were excluded and a further 367 papers were excluded after assessment of the full texts. An additional seven papers were identified through searching relevant manuscript reference lists during the screening stage. In total, 62 studies met the inclusion criteria. Figure 2 outlines each stage of the study selection process.

### Characteristics of included studies

Table 1 summarises the key characteristics and findings from the 62 included studies. Overall, most studies were undertaken in North America (*n* = 20), Europe (*n* = 17) or Australia (*n* = 8), with only few studies undertaken in Asia (*n* = 7). Across the 62 included studies, there were a total of 105,268 children ranging in age from 18 months to 12 years, but the majority of studies (*n* = 45) focused on primary school aged children aged between 5 and 12 years. Seven studies were exclusively in pre-school aged children (< 5 years) [24, 40, 70, 80, 82], while 10 studies involved children spanning a broader age range (from pre-school up to 12 years) [34, 42, 47, 56, 61, 63, 64, 81]. Most studies examined associations between home environments and child body weight cross-sectionally (*n* = 51), with fewer prospective studies (*n* = 11). Most studies examined a single domain (*n* = 41) or two domains (*n* = 16), with only five studies assessing all three pre-defined domains of the HE [78–82].

**Table 1:** Characteristics and results table for included studies (*n* = 62)

Author, year	Study design	Country	Sample N (% male), age	HE Constructs assessed	HE measure (items, method of administration)	Adiposity Outcome	Key finding: Relationship with adiposity
**MEDIA ENVIRONMENT ONLY (STUDIES** ***N*** **= 23)**							
Adachi-Mejia et al. 2007 [16]	Cross	USA	2343 (50%), 9–12 y	Physical - TV in bedroom. TVs in household	2 items; PR	BMI z-scores^a^	TV in bedroom associated with OW (OR = 1.32).
Anderson et al., 2010 [24]	Cross	USA	8550 (51%), 4 y	Social *-* Caregiver limits around screen-time (< 2 h/day)	1 item; PR.	BMI z-scores^a^	Limits on screen-viewing duration associated with lower odds of OB (OR = 0.85, *p* = 0.002).
Atkin et al. 2013 [25]	Prosp	UK	2064, (T0) 9–10 y (45%); (T1) 10–11 y (41%)	Physical *-* Media equipment (TV, computer) in bedroom and household (assessed T0, T1y)	2 items; PR	BMI, weight status (NW/OW)^d^	Children with OW more likely to have a TV in bedroom (T0 and T1) compared to children with NW. No effect of computer in bedroom. Higher total bedroom media score in children with OW compared to NW (T1y).
Borghese et al. 2015 [26]	Cross	USA, Canada	1201 (43%), 10 y.	Physical - TV in bedroom	ISCOLE HNEQ; 1 item; PR.	Body Fat %	Canadian sample: TV in bedroom associated with higher BF% compared to no TV (boys: 21.8% vs 18.1%; girls: 24.9% vs 21.3%). American sample: TV in bedroom associated with higher BF% compared to no TV in boys (21.4% vs 18.9%) only.
Cameron et al. 2013 [27]	Cross	7 European Countries^b^	7234 (48%), 10–12 y.	Physical - TV in bedroom	1 item; PR.	BMI & WC^d^	TV in bedroom associated with higher BMI and waist circumference (in 4/7 European countries).
Chahal et al. 2013 [28].	Cross	Canada	3398 (50%), 10–11 y	Physical - Media equipment in bedroom (TV, DVD player, computer, video game console, phone)	Project EAT-III questionnaire, (5 items), PR and CR	BMI and weight status (NW, OW, OB)^d^	Electronic media devices (3+) in bedroom associated with greater odds of OW (OR = 2.57) or OB (OR = 2.23, *p* < .05) compared to no devices. Increased odds of OB for children with TV in bedroom (OR = 1.64), or computer in bedroom (OR = 1.47). Increased odds of OW for children with phone in bedroom (OR = 1.42).
Chaput et al. 2014 [29]	Cross	Canada	502 (41%), 9–11 y	Physical – Media equipment in bedroom (TV, computer, video games)	ISCOLE HNEQ; 3 items; PR.	BMI z-scores, BMI centile, BF%^e^	2–3 screens in bedroom associated with higher BF% compared to no screen. TV in bedroom associated with higher BF% compared to no TV. Computer in bedroom not associated with BF%.
Dube et al. 2017 [30]	Cross	Canada	2334 (47%), 10–11 y.	Physical - Media equipment in bedroom (TV, computer, video game, tablet, mobile phone)	Project EAT-III Q; 5 items; PR.	BMI^d^	≥1 device in bedroom associated with increased odds of OB (OR = 1.82). Increased odds of OB for those with mobile phone (OR = 1.56, 95% CI: 1.24, 1.98), TV (OR = 2.56), and/or computer (OR = 2.79) in bedroom.
Farajian et al. 2014 [31]	Cross	Greece	4552 (49%), 10–12 y	Physical - Media equipment in bedroom (TV, computer, video game)	2 items; CR	BMI^d^	Having both TV and PC/video game console in bedroom associated with increased odds of OW/OB (OR = 1.41).
Ferrari et al. 2015 [32]	Cross	Brazil	441 (49%), 9–11 y.	Physical - Media equipment in bedroom (TV, computer, video games)	ISCOLE HNEQ;3 items; PR.	BMI^e^	Video games in bedroom associated with higher BMI (β = 0.94). 2–3 electronic devices in bedroom associated with higher BMI (β = 0.51). No association with BMI for TV and computer.
Ferrari et al. 2017 [33]	Cross	Brazil	328 (52%), 9–11 y	Physical - Media equipment in bedroom (TV)	ISCOLE HNEQ; 3 items; PR.	BMI, BF %, WC^e^	No associations with BMI.
Hardy et al. 2012 [34]	Cross	Australia	1141 (50%), 5–12 y	Physical – TV in bedroom. Social - Caregiver rules around screen-time duration.	ASAQ; 4 items; PR; validated.	BMI, weight status (HW, OW/OB^d^	Girls with OW more likely to have a TV in bedroom compared to girls with NW (OR = 2.00) (no association for boys). No association between caregiver media rules and weight status.
Heilmann et al. 2017 [35]	Prosp	UK	12,556 (51%), 7–11 y.	Physical - Media equipment in bedroom (TV)	1 item; PR	BMI, body fat, weight status^d^	TV in bedroom (at age 7) associated with greater RR of having OW at age 11 (RR for boys = 1.21; RR for girls = 1.31) compared to no TV.
Gomes et al. 2015 [36]	Cross	Portugal	580 (58.1%), 9–11 y.	Physical - Media equipment in bedroom (TV, computer, video games)	ISCOLE HNEQ; 3 items, PR.	BMI^e^	Media in bedroom associated with higher BMI (β = 0.26).
Lane et al. 2014 [37]	Cross	Ireland	8568 (48.7%), 9 y	Physical - Media equipment in bedroom (TV, computer, video games, phone)	4 items; PR.	BMI^d^	TV in bedroom (OR = 1.38) and owning a mobile phone (OR = 1.41) associated with increased odds for OW or OB. No association for computer and games console in bedroom.
Li et al. 2014 [38]	Cross	China	497 (51.7%), 8–10 y	Physical - Media equipment in home (games console, computer, DVD)	3 items; PR.	BMI SDS^b^	No media equipment in home associated with lower risk of OB compared to 1–2 devices, specifically DVD players (OR = 0.68) and games consoles (OR = 0.60). No association for computer.
Lehto et al. 2011 [39]	Cross	Finland	604 (48.3%), 9–11 y.	Physical - Media equipment in bedroom (TV, computer, video games)	2 items; CR.	BMI, WC, WHtR^k^	TV in bedroom associated with higher WC (β = 2.30). Computer/video games in bedroom associated with higher WC (β = 1.33)
Sijtsma et al. 2015 [40]	Cross	Netherlands	1670 (53%), 3–4 y	Physical – TV in bedroom. Number of TVs in home.	LRBQ; 2 items; PR.	BMI z-score^i^	TV in bedroom associated with higher BMI. No association between number of televisions in home on and BMI.
Tiberio et al. 2014 [41]	Prosp	USA	213 (45%), 5–9 y	Social - Caregiver monitoring and limit setting around media use	3 items^n^; PR	BMI z-scores^a^	Less maternal monitoring associated with higher BMI z-scores at age 7 (β = −.23, *p* < 0.01) and steeper increases in BMI z-scores from ages 5 to 9 y (β = −.058, *p* < 0.01).
Rutherford et al. 2015 [42]	Prosp	Australia	4983 (51.4%) 4–9 y	Physical – TV in bedroom and computer in home. Social - Caregiver rules around TV viewing duration	3 items; PR	BMI^d^	No associations with BMI and weight status.
Lin et al. 2019 [43]	Cross	Taiwan	1031 (50%), 7–12 y	**Media:** Social – Caregiver modelling and limit setting around screen-viewing	6 items; PR.	BMI^q^	No association with weight status.
Paduano et al. 2020 [44]	Cross	Italy	588 (53.2%), 6–7 y	**Media:** Physical – TV in bedroom	1 item; PR.	BMI z-score^d^	TV in bedroom associated with higher odds of OW/OB (OR = 1.1)
Park et al. 2019 [45]	Cross	USA	129 (48.1%) 2-5y	**Media:** Physical - TV in bedroom	FNPA^p^; 20 items; PR; validated.	BMI z-scores, weight status^a^	No association with weight status.
**FOOD ENVIRONMENT ONLY (*n*** **= 13)**							
Cassimos et al. 2011 [46]	Cross	Greece	335 (54.03%), 9–12 y.	Physical - Availability of and access to sweets and juice in the home.	21 items; PR.	BMI^d^	Availability of sweets associated with increased odds of OW/OB (OR = 0.357).
Chen et al. 2018 [47]	Cross	China	222 (51.5%), 3–6 y	Physical - Energy dense foods at home	FEAHQ; 29 items; PR, validated.	BMI^e^	Availability of energy dense foods associated with higher BMI (β coefficient = 0.30, *p* < .01)
Couch et al. 2014 [13]	Cross	USA	699 (50.2%), 6–11 y	Physical – Availability of energy dense foods and nutrient-dense foods Social – Caregiver modelling positive eating behaviours. Caregiver rules around child eating.	EMS (7 items); FEAHQ (3 items); AWQ (12 items); PR; validated	BMI z-scores^a^	Encouragement/modelling of ‘healthy eating’ negatively associated with BMI z- scores (β coefficient = −0.17). No association between food availability and BMI z-scores. No association between caregiver rules around child eating and BMI z-scores.
Downs et al. 2009 [48]	Cross	Canada	225 (NP), 9–12 y	Physical - Food and beverages in the home.	FAQ; NP; CR interview.	BMI^d^	No associations with BMI.
Humenikova et al. 2008 [49]	Cross	Czech and USA	US: 45 (33%) Czech: 97 (43%), 10–11 y	Physical - ‘*healthful’* foods (e.g. fruits, vegetables, low-fat dairy) in home	Shelf Inventory; 80 items; PR, validated.	BMI percentiles^d^	*America:* No association with BMI z-score. *Czech:* Greater availability of ‘healthful’ foods associated with lower BMI z-scores (r = −.203, *p* < .05).
Gable et al. 2000 [50]	Cross	USA	65 (43%), 6–10 y	Physical aspects - Food and beverages in the home.	FAQ; NP; PR.	BMI^m^	No associations with BMI.
Lopez-Barron et al. 2015 [51]	Cross	Mexico	684 (45.5%), 10–11 y	Physical - Food and beverages in the home.	Food inventory; 13 items; CR.	BMI z-scores, Height z-scores, WC^b^	OW/OB associated with increased odds of availability of fruits and vegetables (OR = 1.10, *p* = 0.035). OW/OB associated with lower availability of energy-dense foods at home (OR 0.56, *p* < .001)
MacFarlane et al. 2009 [52]	Prosp	Australia	T0 = 161 (50%) 5–6 y T1 = 132 (50%) 10–12	Physical - Energy-dense foods at home Social - Caregiver policies around energy-dense snacks and fast foods.	Family food environment; 7 items; PR.	BMI z-scores^a^	No associations with BMI z-scores.
Terry and Beck, 1985 [53]	Cross	USA	16 (56%) 8–12 y	Physical - Foods in the home (foods traffic lighted based on caloric value; number of red, yellow and green foods visible in home)	In-home observation of food environment × 2.	BMI^l^	**Observation 1:** No difference between OB and NW in availability of energy-dense foods. **Observation 2:** No difference between OB and NW in availability of energy-dense foods.
Palfreyman et al. 2014 [54]	Cross	UK	484 (51%), 1–8 y	Social - Caregiver modelling of healthy eating behaviour	PARM; 18 items; PR.	BMI z-scores^j^	No association with BMI.
Van Lippevelde et al. 2013 [55]	Cross	7 European Countries^15^	6374 (47%), 10–12-y.	Physical – Breakfast type foods (milk, cereal, breads) in home	1 item; PR.	BMI z-score^h^	No association with BM.
Vaughn et al. 2017 [56]	Cross	USA	129 (51%), 3–12 y.	Physical - Food and drinks in the home. Social - Caregiver modelling of eating and limit setting around unhealthy food intake	CAFPP; 124 items; PR; validated	BMI centiles and z-scores^k^	No associations between availability with BMI. No association between caregiver modelling or limit setting around unhealthy eating with BMI.
Quah et al. 2018 [57]	Cross	Singapore	511 (52.1%), 5y	Social – Caregiver modelling and support for healthy eating.	CFPQ; 8 items; PR; validated.	BMI z-score^b^	No association with BMI z-score.
**PHYSICAL ACTIVITY ENVIRONMENT ONLY (*n*** **= 5)**							
Chivers et al., 2012 [58]	Prosp	Australia	2868 (NP%), 1–10 y.	Physical – PA equipment at home. Social - Caregiver support of PA by visiting park or playground with child.	NP; PR.	BMI^d^	Cross-sectional: No associations with weight status. Prospective: No associations with weight status.
Sijtsma et al. 2015 [59]	Cross	Netherlands	1554 (50%), 3–4 y	Social - Caregiver modelling of PA behaviour	SQUASH; 11 items; PR.	BMI z-score and WC^i^	No association between caregiver modelling of PA and BMI or waist circumference. Caregiver modelling of PA commuting (e.g. walking) associated with lower BMI Z-score (r = −0.062).
Liszewska et al. 2018 [60]	Prosp	Poland	879 (48%), 6–11 y	Social - Caregiver modelling and support of PA	ARPQ (7 items); Modified CFPQ for PA (31 items); PR; validated	BMI z-scores^b^	Caregiver modelling and support of PA associated with lower BMI z-scores (r = −.070, *p* < .05).
Schalkwijk et al. 2018 [61]	Prosp	UK	6467 (51%), 3–7 y	Physical - Access to garden at home	1 item; PR.	BMI^d^	No access to garden associated with increased odds for OW/OB (OR = 1.35).
Umstattd Meyer et al. 2013 [62]	Cross	US/ Mexico	94 (42%), 6–11 y	Physical - PA equipment at home	16 items; interview PR.	BMI percentiles	No associations with BMI.
**STUDIES ASSESSING TWO DOMAINS OF THE HOME ENVIRONMENT (*n*** **= 16)**							
Hales et al. 2013 [63]	Cross	USA	129 (51%), 3–12 y	**Media:** Physical – Media equipment in home (TV, computer, video games) **PA:** Physical - Availability and access to PA equipment	HomeSTEAD; 1015 items; PR; validated.	BMI percentiles^a^	**Media:** No associations with BMI. **PA:** Greater PA equipment associated with lower BMI (‘adult exercise equipment’; r = − 0.26, and ‘child fixed play equipment’; r = − 0.25 and ‘child portable play equipment’; − 0.23).
Jones, et al. 2009 [64]	Cross	Australia	140 (51%), 2–6 y	**Media:** Physical- TV in bedroom. Social aspects - Caregiver rules around TV **PA:** Social - Caregiver rules around PA	Parenting Styles Q; 9 items; PR	BMI^d^	**Media:** No associations with weight status. **PA:** No associations with weight status.
Sleddens et al. 2017 [15]	Prosp	Netherlands	1694 (51.2%), 5–7 y	**Media:** Social – Caregiver limit setting around screen-based activities **PA:** Social – Caregiver support of child PA	ARPQ; 7 items; PR; validated.	BMI z-score^i^	**Media:** Caregiver policy ‘restriction of sedentary behaviour’ associated with greater increases in BMI z-scores from ages 5 to 7. **PA:** No association with BMI.
Taylor et al. 2011 [65]	Cross	Australia	175 (44%), 7–12 y	**Media:** Social– Caregiver modelling and limit setting around of media use. **PA:** Social - caregiver modelling of PA	Parent Physical Activity Practices Q; 11 items; PR.	BMI z-score^d^	**Media:** No association with BMI. **PA:** No association with BMI.
Mathialagan et al. 2018 [66]	Cross	Malaysia	802 (NP%), 10–12 y	**Media:** Social - Caregiver limit setting around electronic media equipment use. **PA:** Social - Caregiver PA levels	42 items; CR; validated.	BMI^b^	**PA:** No association with weight status. **Media:** Caregiver limits on media use associated with lower child weight status.
Rosenberg et al. 2010 [67]	Cross	USA	116 (52.2%), 5–11 y	**Media:** Physical – Media equipment in bedroom and home (TV, computer, games console) **PA:** Physical - PA equipment at home	Home PA equipment scale (21 items); HEES (14 items); PR.	BMI z-score^a^	**Media:** Electronics in bedroom associated with higher BMI z-score (β coefficient = .17, *p* < .05). TV in bedroom not associated with BMI. **PA:** No association with BMI.
Mihrshahi et al. 2017 [68]	Cross	Australia	3884 (49%), 6–10 y	**Media:** Physical - TV in bedroom. Social - Caregiver rules around screen-time **Food:** Physical – Sugar-sweetened beverages (SSB) at home. Social - Caregiver policies around sweet snacks	5 items; PR.	BMI, WHtR - abdominal obesity^k^	**Media**: TV in bedroom associated with higher odds of OW/OB (OR = 1.74) and abdominal OB (OR = 1.96). No limits on screen-time associated with higher odds of abdominal OB (OR = 1.66). **Food:** Availability of SSB associated with higher risk of OW/OB (OR = 1.51) and higher abdominal OB (OR = 1.50) in unadjusted models. No association with adiposity in fully adjusted models.
Keihner et al. 2009 [69]	Cross	USA	299 (47.8%), 9–11 y	**Media:** Physical *-* TV in bedroom. Social *–* caregiver limit setting around screen-time **Food:** Social *-* Caregiver modelling of energy-dense foods	Food and activity diary, FMTS; 4 items; CR and PR.	BMI z-scores, BMI %tiles^a^	**Media:** No associations with BMI. **Food:** No associations with BMI.
Huynh, et al. 2011 [70]	Prosp	Vietnam	670 (49%), 4–5 y	**Media:** Physical - Media equipment in home (TV, computer, video games, portable devices) **Food:** Physical - Food and beverages in the home.	HOME-SF; validated.	BMI, skinfold thickness^g^	**Media**: No associations with changes in BMI or skinfold thickness over 1 year. **Food:** Availability of ‘healthy foods’ negatively associated BMI (girls only) and skinfold thickness (boys and girls) over 1 year.
Serene et al. 2011 [71]	Cross	Kuala Lumpur	1430 (41.5%), 9–12 y	**Food:** Physical – Foods in home. Social - Caregiver encouragement of healthy eating. **PA:** Social - Caregiver support of PA	Q developed based on CFQ and DASH.	BMI^e^	**Food:** No associations with BMI. **PA:** No associations with BMI
Serrano et al. 2014 [72]	Cross	Puerto Rico	114 (42.1%), 12 y	**Food:** Social - Caregiver encouragement of healthy eating **PA:** Social - caregiver encouragement of PA	Team COOL Survey; 76 items; PR; validated.	BMI^a^	**Food:** No association with weight status. **PA:** No association with weight status.
Moreno et al. 2011 [73]	Cross	USA	233 (47%), 5–12 y	**Food:** Social - Caregiver modelling of healthy eating **PA:** Social - Caregiver modelling of PA	FHBS; 27 items; PR; validated.	BMI z-score^a^	**Composite score:** No association between ‘Parent behaviour’ (caregiver modelling of healthy eating and PA) and child BMI z-scores.
Sirikulchayanonta et al. 2011 [74]	Cross	Thailand	280 children (50%), 8–12 y.	**Food:** Physical - Foods available in home **PA:** Physical – Access to PA equipment/garden	11 items; CR. Composite score of ‘home environment’	BMI age- and sex- specific^f^	**Composite score:** Higher risk ‘home environment’ associated with increased odds of OB (OR = 2.8).
Torres et al. 2014 [75]	Cross	Puerto Rico	114 (43%), 12 y	**Food:** Physical - Foods in home. **PA:** Physical - PA equipment at home	Home Physical Environment; 10 items; CR	BMI percentiles^a^	**Food:** Availability of ‘unhealthy’ foods associated with higher BMI (*r* = − 0.25). No association between availability of healthy foods with BMI. **PA:** Access to PA equipment associated with higher BMI (*r* = 0.25).
Crawford et al. 2012 [76]	Cross	Australia	491 (47%), 5–12 y	**Media:** Physical - TV in bedroom and home. Social - Caregiver limit setting around electronic media **PA:** *Physical -* PA equipment at home. Social - Caregiver support of PA	Home environment questionnaire; 46 items; PR; validated.	BMI z-scores^a^	**Media:** TV in bedroom associated with higher BMI z-scores (B coefficient = 0.24). No association between caregiver limit setting and BMI. **PA:** No associations with BM
Vaughn et al. 2019 [77]	Cross	USA	129 (51%), 3–12 y	**Media:** Social – Caregiver modelling and limit setting around electronic media use. **PA:** Social - Caregiver modelling and support of PA	HomeSTEAD; 196 items; PR; validated.	BMI percentile^a^	**Media:** No association with BMI percentile. Caregiver modelling of video games and computer associated with higher BMI percentile (*r* = 0.15). **PA:** Caregiver encouragement of PA associated with lower BMI percentile (*r* = − 0.25). Lack of caregiver support for PA associated with higher BMI percentile (*r* = .17). No association for modelling of PA.
**STUDIES MEASURING ALL THREE DOMAINS OF HOME ENVIRONMENT** (*n* = 5)							
Rodenburg, G., et al. 2013 [78]	Cross	Netherlands	1480 (50.5%), 8–12 y	**Food:** Physical - Food and beverages in the home. Social - Caregiver modelling and support for healthy eating **Media:** Physical – Media equipment (TV, computer) in bedroom. Social – Caregiver modelling of TV viewing. Caregiver rules around screen viewing. **PA:** Physical - PA equipment at home. Social - Caregiver modelling of PA	Home Environment Survey (HES); 84 items; PR; validated. Five composite scores created.	BMI z-score^i^	**Composite score:** ‘Diet- and activity-related positive modelling’ (caregiver modelling of healthy eating, modelling of sedentary behaviour, caregiver snack intake and access to PA equipment) was positively associated with child BMI z-scores (B coefficient = 0.08, *p* < 0.05). No association between ‘High visibility and accessibility to screens and unhealthy food’ and BMI z-scores.
Ihmels et al. 2009 [79]	Cross	USA	854 (51.3%), 6–7 y	**Food:** Social - Caregiver modelling of healthy eating behaviour **Media:** Physical - TV in bedroom. Social - Caregiver monitoring of TV **PA:** Social - Caregiver modelling and support of PA	FNPA; 21 items; PR; validated.	BMI, BMI percentiles^a^	**Food:** Caregiver modelling of healthy eating associated with lower BMI (*r* = −.132). **PA:** Caregiver modelling of PA associated with lower BMI (r = −.086, *p* < .01). **Media:** TV in bedroom associated with higher BMI (r = −.156, *p* < .001). No association between caregiver monitoring and child BMI.
Kim et al. 2014 [80]	Cross	South Korea	241 (47.7%), 2–5 y	**Food:** Social - Caregiver modelling of healthy eating **Media:** Social - Caregiver modelling of media use and limit setting **PA:** Social - Caregiver modelling and support for PA	ACTS; 7 items; PA and healthy eating barriers; 9 items; PR; validated.	BMI^k^	**Food:** Caregiver modelling of ‘healthy eating’ associated with higher odds of OB (OR = 1.11). **PA:** No associations with weight status. **Media:** Caregiver modelling of media use associated with higher odds for OB (OR = 1.01). Caregiver limit setting of media use associated with lower BMI (− 0.12, *p* < 0.05).
Gubbels et al. 2011 [81]	Prosp	Netherlands	2026 (51.2%), 5–7 y	**Food:** Social - Caregiver support for healthy eating **Media:** Social - Caregiver limit setting around electronic media use **PA:** Social - Caregiver support and encouragement of child PA	Modified CFQ for food (9 items); ARPQ (9 items); PR; validated.	BMI z-score^e^	**Food:** Caregiver support/encouragement of ‘healthy eating’ at age 5 associated with lower BMI z-scores at age 7 (B coefficient = 0.07). **PA:** No association with BMI. **Media:** Caregiver limits of media use at age 5 associated with higher increases in BMI from age 5 to 7 (B coefficient = 0.06).
Schrempft et al. 2015 [82]	Cross	UK	1096 (49%), 4 y	**Food:** Physical – Food and beverages in the home. Social - Caregiver modelling of eating **Media:** Physical – Media equipment in home and bedroom. Social– Caregiver modelling and limit setting. **PA:** Physical – PA equipment at home. Social - Caregiver modelling/support of PA	HEI; 32 constructs; CATI PR; validated.	BMI z-score^c^	**Food:** No association with BMI. **Media:** No association with BMI. **PA:** No association with BMI. **Overall obesogenic risk:** No association with BM.

Footnotes:

NB: The measure used examines non-HE related aspects. Only some items from the named measure are relevant to the HE domains examined in this review. Therefore, only the number of items from the measure that were utilised are listed.

^a^Centre for Disease Control and Prevention (CDC, 2000) Growth Reference Charts;

^b^World Health Organisation (WHO) 2007 Child Growth Reference values

^c^UK Growth Reference 1990

^d^International Obesity Task Force (IOTF);

^e^WHO Growth reference (2006)

^f^INMU Thai Growth program as weight for height (WFH)

^g^Asian Population Criteria

^h^WHO Anthro-Plus (2009)

^i^Dutch population in 1997

^j^Child Growth Foundation 1996

^k^Not provided

^l^National Centre for Health Statistics

^m^NHANES I

^n^Capaldi, DM.; Pears, KC.; Wilson, J.; Bruckner, L. Parent Interview. Unpublished instrument. Oregon Social Learning Center; Eugene: 1998

^o^7 European countries are Belgium, Greece, Hungary, Holland, Norway, Spain, Slovenia

^p^The FNPA was used to create a composite score of the home “family obesogenic environment” which was associated with lower BMI z-score (β = − 0.069 (0.032), *p* < 0.05). However this score incorporated several aspects of family life outside the scope of this review. Therefore only the findings relevant to the current review are presented here

^q^Health Promotion Administration, Ministry of Health and Welfare (2013)

*Abbreviations*: *N* cohort size, *SES* Socioeconomic status, *HE* Home environment, *OW* Overweight, *OB* Obese, *BMI* Body mass index, *WHtR* Waist to height ratio, *PA* Physical activity, *NS* Not stated, *Q* Questionnaire, *WC* Weight circumference, *SSBs* Sugar sweetened beverages, *OR* Odds ratio, *TV* Television, *HOME-SF* Home observation for measurement of the environment short form, *CATI* Computer-assisted telephone interviewing, *ENERGY* EuropeaN Energy balance research to prevent excessive weight gain among youth project, *SPEEDY* Sport, physical activity and eating behaviour: environmental determinants in young people, *ISCOLE* International study of childhood obesity, lifestyle and the environment, *HNEQ* Home & Neighbourhood Environment Questionnaire, *GECKO* Groningen expert center for kids with obesity, *HomeSTEAD* Home self-administered tool for environmental assessment of activity & diet family food practices survey, *DASH* Determinants of adolescents’ social well-being and health, *COOL* Controlling overweight and obesity for life, *SQUASH* Short questionnaire to assess health enhancing physical activity, *NIK* Neighbourhood impact on kids; project EAT-III (eating and activity in teens and young adults)-III questionnaire, *FEAHQ* Family eating and activity habits questionnaire, *PARM* Parental modelling of eating behaviour scale, *ACTS* Activity support scale for multiple groups, *HEI* Home environment interview, *EMS* Encouragement and modelling scale, *ARPQ* Activity related parenting questionnaire, *CAFPP* Comprehensive assessment of food parenting practices, *HEES* Home electronic equipment scale, *LRBQ* Lifestyle-related behaviour questionnaire, *ASAQ* Adolescent sedentary activity questionnaire, *FAQ* Food availability questionnaire, *FHBS* Family health behaviour scale, *AWQ* Active where parent-child questionnaire, *ARPQ* Activity related parenting questionnaire, *FNPA* Family Nutrition & Physical Activity screening tool, *FMTS* Food modelling telephone survey

Most studies were published in the 5 years (*n* = 23; 37.1%) or 5–10 years (*n* = 30; 48.4%) prior to this review. Fewer studies had been published before 2009 (*n* = 9; 14.5%), with the earliest study published in 1985. A summary of the association between the food, PA and media domains and child adiposity are presented in Table 2 and Additional files 4, 5 and 6**.**

**Table 2:** Summary of cross-sectional associations between food, physical activity and media domains and child adiposity outcomes (*n* = 58^a^)

Constructs assessed	N (%) studies examining construct	Association with child adiposity outcome		
		Positive association	Negative association	Null association
**Media domain**				
Greater availability & access to electronic devices	29	21	0	8
Caregiver rules/ limits around media	16	2	5	9
Caregiver modelling of media use	5	2	0	3
**Food domain**				
Greater availability & access to EDF	12	3	1	8
Greater availability & access to F&V	11	1	2	8
Caregiver modelling of eating	10	1	3	6
Caregiver rules/ limits around unhealthy eating	3	0	0	3
**Physical activity domain**				
Greater availability of & access to PA	7	1	2	4
Caregiver modelling & support of PA	13	0	4^b^	9^b^

*Abbreviations*: *EDF* Energy dense foods, *F&V* Fruit and vegetables, *PA* physical activity

^a^Four studies [73, 74, 78, 82] are omitted from this table as it was not possible to summarise the findings of studies that created composite scores across two or three domains of the HE. Details of these studies can be found in Table 1

^b^One study [59] examining caregiver modelling of PA reported different findings by the type of activity modelled: Modelling of commuting to school/work by bike or walking was associated with lower BMI Z-score (r = − 0.062) but modelling of vigorous PA was not associated with BMI z-score or waist circumference

### Characteristics of HE and adiposity measures

There was substantial heterogeneity in the measurement tools used to examine the HE. Measurement methods varied, including comprehensive measures of all three domains of the HE such as the Home Environment Interview [82] and Family Nutrition & Physical Activity Screening Tool [79], and measures of one or two domains of the HE such as the Family Eating and Activity Habits Questionnaire [47], the Family Food Environment Questionnaire [52], and The Home Self-Administered Tool for Environmental Assessment of Activity and Diet (HomeSTEAD) [63]. Other measures were less comprehensive, using single items or a limited number of items to measure only one aspect of the HE (e.g. availability of television in the bedroom). Most studies exclusively used caregiver and/or child self-completed (*n* = 49) or interviewer-delivered (n = 4) questionnaire methodology. Few studies (n = 4) used in-home observation, and this was generally used in addition to questionnaire-based measures.

Measures of adiposity were taken via trained researcher (*n* = 48), parent-report (*n* = 5) or a combination of both (*n* = 2). Most studies (*n* = 61) used height and weight measurements to derive BMI, BMI z-score or BMI percentile. These measurements were taken using a calibrated weighing scale and stadiometer (*n* = 53) or with parents own scale and measuring tape (*n* = 9). Only nine studies supplemented height and weights with additional adiposity measures, such as body fat percentage (BF%) (*n* = 1), skinfold thickness (*n* = 1), Fat Mass Index (n = 1), waist circumference (*n* = 3), waist-to-height ratio (WtHR) (*n* = 1), or a combination of these (*n* = 2). Only one study used BF% as the primary outcome (*n* = 1).

### Relationship between home environment and adiposity outcomes

#### Media domain

Twenty-nine studies examined physical aspects of the home media environment, with most studies (21/29; 72.4%) demonstrating positive associations between availability and access to electronic media equipment in the home and measures of child adiposity. This association was observed across children aged 3–12 in both cross-sectional (*n* = 19) [16, 26–32, 34–40, 44, 67, 68, 76, 79] and longitudinal studies (*n* = 2) [25, 35]. One large prospective study of 12,556 children from the U.K. reported that having a TV in the bedroom at age 7 was associated with increased risk of children developing overweight at ages 7 and 11, compared to those without a TV in their bedroom [35]. Similar results were reported in another prospective study following UK children from ages 9/10 to 10/11 (*n* = 2064) [25]. Over the past decade, there have been considerable changes in children’s use of screens, with a decline in TV viewing and an increase in use of other devices (e.g. tablets, mobile phones, laptops) to access video content online [83]. As such, more recent studies have expanded the scope of their measurement tools to capture a broader range of electronic devices in the home (e.g. games console, mobile phones, tablets, laptops etc.). These studies suggest that the number of electronic devices, as well as the types of devices available may have implications for child weight, with a greater number of electronic devices present in the home associated with higher BMI z-scores in children aged between 9 and 12 [29–32, 37, 38]. A study of children aged 9–11 (*n* = 502) reported that those with multiple devices (2–3 or more) in their bedroom had higher body fat percentage than children with no devices in their bedroom [29]. The remaining studies (8/29; 27.6%) reported null associations between availability of media equipment and child adiposity [33, 42, 45, 63, 64, 69, 70].

Sixteen studies examined relationships between social aspects of the home media environment and child adiposity [15, 24, 34, 41–43, 63–66, 68, 69, 76, 79–81]. Of these, five studies measured caregiver modelling of media use [43, 64, 65, 77, 80]. Two studies conducted in children aged 2–5 years [80] and 3–12 years [77] reported positive associations between caregiver modelling of media use and child BMI. The remaining three studies, involving children aged 2–6 years [64], and 7–12 years from Australia [65] and Taiwan [43], found no associations between caregiver modelling of media use and child adiposity. Sixteen studies examined relationships between caregiver rules and limit setting around media use and child adiposity, with inconsistent findings [15, 24, 34, 41–43, 63–66, 68, 69, 76, 79–81]. Four cross-sectional studies reported associations between fewer caregiver limits and less monitoring of media use with higher risk for abdominal obesity in Australian primary school aged children (aged 6–10 years, *n* = 3884) [68] and higher BMI scores in Malaysian children aged 10–12 (*n* = 802) [66], and preschool aged children from the US (*n* = 8550) [24] and South Korea (*n* = 241) [80]. One longitudinal study from the US (*n* = 213) reported similar findings with less caregiver monitoring of media use predicting steeper increases in children’s BMI z-scores from ages 5 to 9 [41]. However, two prospective studies from the Netherlands which pooled data from the Dutch KOALA birth cohort (*n* = 1694 and *n* = 1819) found the reverse relationship, with limits on electronic media use associated with greater increases in child BMI z-scores from ages 5 to 7 [15, 81]. Nine studies (56.3%) reported no relationships between caregiver rules and limits around media use and child weight [34, 42, 43, 56, 65, 69, 76, 79, 84]. Findings are summarised visually in Additional file 4**.**

#### Food domain

Fifteen studies examined relationships between physical aspects of the home food environment and child adiposity, demonstrating inconsistent findings. Six studies reported associations between food or drink availability and access, and child adiposity [46, 47, 49, 51, 70, 75]. Greater availability of nutrient dense foods (e.g. fruits and vegetables) were associated with lower BMI- z-scores in Czech 10 to 11 year olds (*n* = 97) [49], while higher availability and access to energy-dense foods (e.g. SSBs, sweets, etc.) predicted higher BMI among Chinese 3–6 year olds (*n* = 222) [47] and Greek children aged 9 to 12 years (*n* = 335) [46]. But conversely, one study of Mexican children (*n* = 684) aged 10–11 reported the opposite relationship; children with OW/OB had greater access to fruits and vegetables and less access to energy-dense foods (e.g. confectionary items, cookies, SSB and salted snacks) in the home [51]. Eight studies exploring food availability and accessibility in the home with measures of child adiposity found no relationship between them [13, 48, 50, 52, 53, 55, 71].

In total, eleven studies assessed relationships between social aspects of the home food environment and child adiposity, but these varied in their scope. Eight assessed caregiver modelling and/or support of eating [54, 57, 69, 71, 72, 79–81], one assessed caregiver rules and limit setting around food [68] and two assessed both caregiver modelling of eating and caregiver rules/limit setting [13, 56]. Of the ten studies examining the role of modelling, three demonstrated associations between caregiver modelling and/or support of healthy food intake and lower child BMI z-scores; this was observed both cross-sectionally in U.S. children aged 6–11 (*n* = 699) and aged 6–7 (*n* = 854) [13, 79] and longitudinally in Dutch children followed from ages 5 to 7 [81]. Contrastingly, one cross-sectional study conducted in South Korean preschool children (*n* = 241; 2–5 years) found greater modelling of healthy eating was associated with higher child BMI [80]. Null associations between caregiver modelling and/or support of food intake and child adiposity were observed in the remaining six studies [54, 56, 57, 69, 71, 72, 76]. A further three studies assessed caregiver rules and policies around unhealthy eating and found null associations with child adiposity across all studies [13, 56, 68]. The findings are summarised in Additional file 5**.**

#### Physical activity domain

Seven studies examined relationships between the home PA environment and measures of child adiposity. Two cross-sectional studies reported negative associations between availability and access to PA equipment and child adiposity outcomes [61, 63]. A large UK-based study (*n* = 6467) of 3–7 year olds reported children with access to garden space had lower odds of OW/OB compared to those without garden access [61]. These findings are consistent with a US study of children aged 3 to 12 years which found both the amount and condition of the PA equipment available in the HE was associated with lower BMI percentile [63]. Conversely, one study (*n* = 114) of 12 year olds in Puerto Rico reported greater access and availability of recreational and sports equipment at home was associated with higher child BMI [75]. The remaining three cross-sectional [62, 67, 76] and one longitudinal [58] study all reported null associations between access and availability of PA equipment and child BMI.

Thirteen studies explored social PA environments in the home in relation to child adiposity. Two studies, a cross-sectional study of 854 children aged 6–7 from the U.S. and a longitudinal study of 879 Polish children aged 6–11 reported caregiver modelling and support of PA were associated with lower child BMI z-scores [60, 79]. In contrast, a study of pre-schoolers from the Netherlands (*n* = 1554) found relationships with child adiposity varied according to the type of activity modelled by the parent. Higher levels of active travel (e.g. commuting to work via walking or bike) were associated with lower BMI z-scores in children aged 3–4 years old, but there were no associations for other types of modelled vigorous activity (e.g. running) [59]. One cross-sectional study examined caregiver modelling, support and encouragement of PA as separate constructs and found variation in the association with adiposity outcome; with null associations observed for caregiver modelling of PA, while encouragement and support of PA were associated with lower BMI percentiles [77]. The remaining nine studies reported null associations between caregiver modelling and/or support of children’s PA and measures of adiposity [15, 58, 65, 66, 71, 75, 76, 80, 81]. Findings are summarised in Additional file 6.

#### Composite scores of multiple domains of home environment

Composite scores of the HE reflect the combined contribution of more than one of the pre-defined domains – food, PA or media - within the HE. In this review, we identified four studies which generated composite scores across two (*n* = 2) or three (n = 2) domains of the HE and examined associations with child adiposity [73, 74, 78, 82], with mixed findings. One cross-sectional study of U.K. children aged 4 years (*n* = 1096) developed a composite score for the overall physical and social aspects of the HE, and for each of the pre-defined domains – the food, PA and media environment. No relationship with child BMI z-scores were observed for either the overall HE composite score, or any of the food, activity and media domains [82]. A second study, conducted in children aged 8–12 in Bangkok (*n* = 280), created a composite of the physical food and PA environments. Composite scores indicating a ‘lower quality’ (i.e. more obesogenic) HE were associated with 2.8 times higher risk of child obesity [74]. A third cross-sectional study of U.S. children aged 5–12 (*n* = 233) took a slightly different approach, developing a composite score called ‘Parent behaviours’ which assessed caregiver modelling of healthy eating and caregiver modelling and support of PA. No associations with child BMI z-scores were observed [73]. The fourth cross-sectional study of children aged 8–12 from the Netherlands (*n* = 1480), examined physical and social aspects of the HE by combining items into clusters capturing caregiver practices relating to food, PA and media-related energy balance behaviours. The study reported a weak positive association between ‘diet- and activity-related modelling’ and child BMI z-scores, suggesting that children with parents who exhibit greater modelling of healthy eating, less sedentary behaviour and who live in a home with greater availability of PA equipment actually had higher BMI z-scores [78]. Null associations were observed for the remaining four constructs (‘low availability of unhealthy food’, ‘High visibility and accessibility of screens and unhealthy food’, ‘diet and activity related positive modelling’, ‘positive modelling on sports and fruit’) and BMI z-scores. As these studies utilised a composite scoring system, it was not possible to establish the independent contribution of individual aspects of the HE on weight.

### Risk of bias

Overall, 38/62 (61.3%) of the identified studies were rated as high quality based on the NOS quality assessment criteria. The most common methodological weaknesses were in selection and comparability of studies; with 37/62 of studies (59.7%) providing inadequate justification of sample size, 49/62 (79%) providing an inadequate description of response rate or lack of comparison between respondents/non-respondents, and 22/62 (35.5%) failing to control for important confounding factors such as age, sex, SES, energy balance behaviours or parental adiposity. Full details and individual study scores are described in Additional file 3**.**

## Discussion

This is the first systematic review to appraise and synthesise the evidence for associations between the physical and social aspects of the food, PA and media domains of the HE with measures of adiposity in childhood (≤12 years). The most consistent associations were observed for the physical aspects of the home media environment, with greater availability and access to electronic media devices in the home, and specifically in the child’s bedroom, associated with higher risk of adiposity (21/29 studies). Findings were less consistent for the smaller number of studies examining physical aspects of the home food or PA environments. Half (8/15) of the studies examining physical food environments reported null associations, while similar numbers (6/15) demonstrated positive associations between more obesogenic food environments and higher child adiposity. Findings were similarly mixed for PA environments; with 4/7 reporting null associations, 2/7 reporting negative associations and 1/7 study reporting positive associations between access to physical activity equipment/garden space and adiposity. Fewer studies assessed social aspects (e.g. caregiver modelling or limit setting) of the home environment in relation to child adiposity and findings were again mixed; 9/16 media environment, 7/11 food environment and 9/13 physical activity environment studies reported null associations with child adiposity outcomes.

Although research has shown that children learn behaviour from those around them [7, 19, 85], we found limited evidence that behaviours learned at home translate into child weight outcomes. Caregiver modelling of behaviours, across the food, activity and media domains of the HE, were not consistently associated with child adiposity measures in expected directions. The variation in findings may in part result from a lack of consensus in how these constructs are defined and measured. Some studies defined modelling simply as how often parents consumed specific foods or beverages, or the length of time parents spent engaging in activities (e.g. PA or screen-based activities). This approach fails to consider a fundamental aspect of modelling - the frequency with which a child observes these behaviours. Additionally, associations with adiposity outcomes were largely explored cross-sectionally [64], hindering understanding of the directionality of associations, and failing to capture variations over time depending on children’s age, family circumstances. One of the few studies to examine the prospective relationship between social aspects in the HE (e.g. caregiver modelling, caregiver rules/limit setting) and adiposity, revealed that caregiver encouragement of ‘healthy eating’ at age 5 was associated with lower BMI z-scores at age 7 and caregiver restriction of sedentary time at age 5 were associated with higher increases in BMI from age 5 to 7 [81]. Importantly, this study was one of the first to simultaneously examine the influence of multiple aspects of the social HE. Such holistic approaches are important to incorporate a range of factors potentially contributing to child weight development.

Over half of the studies included at least one measure of the home media domain and it was the aspect of the HE most consistently linked to child weight outcomes. This is perhaps unsurprising as greater availability of media in the home has been shown to be associated with weight-related energy balance behaviours; increased sedentary behaviour, decreased activity levels and increased snacking [86]. The more consistent relationship between child adiposity and the physical home media environment may partly result from the fact it is more stable, less complex, and therefore easier to characterise and measure than the home food environment. Unlike the food environment, the media environment is unlikely to fluctuate from day-to-day or vary with seasonal changes to the same extent. It is also arguably easier to report the number and location of electronic devices in the home, than of food and beverage products. Drawing on this point, studies examining availability of foods and beverages in the home were cross-sectional, collecting data at a single time point. This approach fails to capture fluctuations in the types and/or amount of foods and beverages available in the home over the course of days, weeks and months. Thus, the foods available at data collection may not reflect the foods that are typically available within the home [11]. It is important for studies to account for this variation in the measurement. The general lack of longitudinal studies identified in this review also means we cannot conclude if features of the HE are driving excess weight gain in children or whether any observed associations result from parents modifying HEs in response to their child’s weight status (or weight related behaviour). For example, parents of children with higher adiposity may reduce the availability of energy dense foods at home in an attempt to improve their child’s dietary intake and achieve a healthier weight status.

Another possible explanation for the heterogeneity in findings across studies may be due to variation in the degree of adjustment in statistical models. Seven out of fifteen studies examining associations between food availability and child adiposity failed to include important potential confounding variables (e,g, SES or parental adiposity) in the statistical model. Controlling for such confounding variables is important to understand the true association between aspects of the HE and adiposity. This fact is highlighted in the results of a large study of 6–10 year olds in Australia (*n* = 3884) which reported results based on unadjusted and adjusted models. In the unadjusted model, greater availability of SSBs was associated with higher odds of OW or OB and abdominal obesity [68]. Conversely, in the statistical model adjusted for age, sex, SES and meeting recommended PA levels, no association with adiposity was observed. Varying degrees of adjustment in the included studies may partially account for variation in findings between studies.

Despite the arguably more straightforward composition of the home media environment, the measures utilised by studies included in this review were limited in scope and rarely captured the diversity of electronic devices currently available to children [87]. Most studies (*n* = 16/29) focussed on availability and access to televisions and/or computers within the home, perhaps reflecting the fact that growth in commercially available electronic devices is relatively recent. Ofcom figures reveal U.K. electronic device ownership and use has increased substantially over the last decade, with tablet ownership among 5–15 year olds, rising from 2% in 2011 to around 50% in 2018 [83]. The most comprehensive measure of the media environment was utilised by Canadian researchers, Dube et al. [28] and Chahal [30], who collected data in a cohort of children aged 10 to 11 (*n* = 2334 and *n* = 3398 respectively). Home availability of multiple electronic devices, including TVs, DVD players, computers, video game consoles, tablets and cell phones, were positively associated with higher child weight status [28, 30]. As home media use continues to evolve, it is important for future research to capture the increasing diversity of electronic devices available, along with use of different media platforms, when exploring the impact of the home media environment on children’s weight development.

Evidence for relationships between the home PA environment and child adiposity was very inconsistent. Most studies reported null associations with adiposity for the social PA environment (caregiver modelling and support of PA), while findings for associations between the physical PA environment (availability of and access to PA equipment) and adiposity varied. It is possible that the home PA environment is less important than the home media environment for influencing energy balance and thus weight in childhood. The home media environment has been found to contribute significantly towards sedentary behaviour [12], and energy intake [86], and was more consistently associated with weight in this review. The relationship between PA and child adiposity is complex, reviews have generally found an absence of convincing evidence for the contribution of PA to child adiposity [88, 89], which may in part be due to methodological weaknesses and imprecise measurement of PA levels. Excess weight gain has also been linked to a reduction in physical activity levels further complicating the relationship [90–92]. In addition, there may be age-related variation in the relationship between the home PA environment and child adiposity. The home PA environment may be more influential for younger children, who spend more of their time in the home setting. A large UK-based study (*n* = 6467) of 3–7 year olds reported that children with access to garden space had lower odds of OW/OB compared to those without garden access [61]. In contrast, as children reach secondary school age and gain independence, the neighbourhood and school activity environments may play a greater role in shaping energy balance behaviours and adiposity. More research measuring multiple components within the HE and in different age groups is needed to establish how and in what ways the home food, PA and media environments may interact to influence a child’s weight development.

Across all HE domains there was a notable lack of consensus on how to define and measure the HE. As a result, very few studies used validated measures (*n* = 20/62). Additionally, few studies attempted to characterise the holistic obesogenic HE, instead focusing on individual aspects of a single domain and often using one or two items to measure a single construct (*n* = 13). Studies that utilised validated measures (n = 20) tended to be more comprehensive (e.g. 15,36,96), however it should be noted this did not always result in clearer relationships with child weight outcomes [81, 82]. For example, Schrempft et al. [82] used a validated measure to comprehensively examine the three pre-defined domains of the HE but failed to ascertain associations with adiposity.

## Limitations and recommendations for future research

This review is not without limitations. It was restricted to studies published in English-language, and non-clinical, non-intervention studies. As such we excluded studies in which the population received some type of intervention, for example federal support schemes (e.g. Head Start), which likely limited the number of studies included in the review and limited the number of low-income populations. Although interventions are important for determining causal relationships, this review focussed on observational studies as it is important to understand the effect of ‘real-world’ home environments before deciding how and where to intervene.

There are several problems with the current evidence base, limiting the conclusions of this review. The majority of included studies (*n* = 51/62) were cross-sectional. As discussed, the lack of longitudinal research (11/62 studies) means it is not possible to uncover the direction of relationships between the HE and child adiposity or infer causality. Prospective studies from birth with measurement at multiple time points are needed to identify which aspects of the HE promote or protect against excess weight gain in childhood. Ultimately robust randomized controlled trials of intervention studies will be necessary to determine which aspects of the home environment can be effectively modified to reduce excess weight gain in childhood.

The majority of included studies also predominantly relied on caregiver report and were thus susceptible to social desirability biases [93]. This may present a particular problem when exploring associations with child adiposity if reporting bias varies by weight status. For example, parents of children with overweight or obesity may be more likely to underreport availability of energy dense food in the home. However, caregiver report measures remain the best method for collecting HE information at scale, and they have been shown to be validated using objective measures (e.g. wearable cameras) [94].

There is a lack of research conducted in minority ethnic groups and low socioeconomic status (SES) families (only 15/62 studies (24.2%) considered differences in HE by SES). SES may well confound or moderate the relationship between the HE and child adiposity. For example, in economically developed countries, low SES households are more likely to have electronic devices available in the child’s bedroom compared with higher SES households [16, 95]. Little is currently known about the HE in low-income countries, or about how social inequalities influence the overall obesogenic nature of the home and how this in turn may influence children’s weight development. Alongside differences by age-groups and SES, future research should also consider individual variation in susceptibility to obesity. Not everyone interacts with the obesogenic environment in the same way [96]; for example, availability of energy dense foods may only be associated with increased adiposity in children with an avid appetite. Individual differences in susceptibility to an obesogenic environment likely influence associations between the HE and child adiposity.

Finally, heterogeneity in the measures used and a lack of consensus in both language and definitions of constructs means comparison of findings across studies is impeded. This review emphasises the need to harmonize definitions and measurement of the HE, in order to gain a reliable understanding of how factors within the home contribute to adiposity in childhood, and ultimately inform targeted family obesity prevention and treatment programs.

## Conclusion

This review suggests that the most robust associations between the HE and child adiposity are observed within the physical home media environment. It is not clear whether this is due to a stronger relationship between the media environment and child weight development, compared to the food or PA environments, or whether it is an artefact of it being the HE domain most frequently investigated, and most accurately captured, in current research. This review also highlights that despite the large number of studies identified, there is a lack of agreement on how to conceptualise and measure salient aspects of the HE hypothesised to relate to health outcomes. Consensus is needed for a ‘gold standard’ measurement of the multidimensional HE. Future research should focus on utilising comprehensive measures of multiple HE domains in order to understand how, and to what extent, the different aspects of the HE interact to influence children’s weight development. Such efforts would facilitate the development of evidence-based guidance on how best to modify the HE to reduce childhood obesity risk.

## Supplementary Information

**Additional file 1:.** PRISMA Checklist

**Additional file 2:.** Search strategy used for Medline Based on the PICOS framework.

**Additional file 3:.** Risk of bias assessment using the Newcastle Ottawa scale (NOS) for cohort studies included in home environment systematic review (Adapted for the assessment of observational, cohort studies (Modesti et al. [23]).

**Additional file 4:.** Cross-sectional association between physical and social aspects in the home media domain and child adiposity outcomes.

**Additional file 5:.** Cross-sectional association between physical and social aspects in the home food domain and child adiposity outcomes.

**Additional file 6:.** Cross-sectional association between physical and social aspects in the home PA domain and child adiposity outcomes.

## Data Availability

Data generated as part of this systematic review are included as supplementary material. Additional information may be available upon request.

## Abbreviations

HE: Home Environment
PA: Physical Activity
SSB: Sugar Sweetened beverages
PRISMA: Preferred Reporting Items for Systematic Reviews and Meta-analysis
PROSPERO: International Prospective Register of Systematic Reviews
BMI: Body Mass Index
NOS: Newcastle Ottawa Scale
SES: Socioeconomic Status
HomeSTEAD: Home Self-Administered Tool for Environmental Assessment of Activity and Diet
OB: Obese
OW: Overweight
