# Supplementary material for: The relationship between the home environment and child adiposity: a systematic review
Source: Int J Behav Nutr Phys Act. 2021 Jan 6;18:4. doi: 10.1186/s12966-020-01073-9 (PMC7788808; doi:10.1186/s12966-020-01073-9)
Supplement: Supplementary file 2 — Additional file 2:. Search strategy used for Medline Based on the PICOS framework. [file 12966_2020_1073_MOESM2_ESM.docx]

**Additional File 2:** Search strategy used for Medline Based on the PICOS framework.

| 1 | exp family/ |
| --- | --- |
| 2 | exp child/ |
| 3 | exp PARENTS/px |
| 4 | (child* or toddler* or infant*).mp |
| 5 | (boy* or girl* or youth*).mp |
| 6 | (schoolchild* or school child* or school-child*).mp |
| 7 | (pediatr* or paediatr*).mp. |
| 8 | (preschool* or pre-school*).mp. |
| 9 | 12 year old*.mp. |
| 10 | age 12.mp. |
| 11 | 1 or 2 or 3 or 4 or 5 or 6 or 7 or 8 or 9 or 10 |
| 12 | home environment.mp. |
| 13 | (home media or home media environment or tv viewing).mp |
| 14 | (obesogenic environment or obesogenic home or unhealthy home).mp |
| 15 | (home food environment or food environment or family meal* or family mealtime* or family meal-times or food availability or home food or available food or food access* or food in home or parental monitoring).mp |
| 16 | (home activity or physical activity home or home physical activity or sedentary behaviour or parental activity or family physical activity or physical activity social environment or parent physical activity modelling or physical activity or physical home environment).mp |
| 17 | (screen time or media physical environment or screen-time or media social environment or sedentary behaviour).mp |
| 18 | (parental policies or parental role model or parental modelling).mp |
| 19 | (snacking or eating behaviour or food intake).mp. |
| 20 | 12 or 13 or 14 or 15 or 16 or 17 or 18 or 19 |
| 21 | (weight or bmi or body mass index).mp |
| 22 | (growth charts or Anthropometr*).mp. |
| 23 | (BMI z-scores or BMI-for-age or weight-for-length percentiles or weight-for-height percentiles or waist circumference).mp. |
| 24 | (adipos* or weight status).mp. |
| 25 | exp child development/ |
| 26 | exp body weight/ |
| 27 | exp obesity/ |
| 28 | exp childhood obesity/ |
| 29 | exp body mass index/ |
| 30 | 21 or 22 or 23 or 24 or 25 or 26 or 27 or 28 or 29 |
| 31 | 11 and 20 and 30 |
| 32 | limit 31 to (english language and humans and ("all infant (birth to 23 months)" or "newborn infant (birth to 1 month)" or "infant (1 to 23 months)" or "preschool child (2 to 5 years)" or "child (6 to 12 years)") and journal article) |
| *The syntax used in this search strategy was adjusted where necessary according to the requirements of each database. | |
