# Supplementary material for: The relationship between the home environment and child adiposity: a systematic review
Source: Int J Behav Nutr Phys Act. 2021 Jan 6;18:4. doi: 10.1186/s12966-020-01073-9 (PMC7788808; doi:10.1186/s12966-020-01073-9)
Supplement: Supplementary file 3 — Additional file 3:. Risk of bias assessment using the Newcastle Ottawa scale (NOS) for cohort studies included in home environment systematic review (Adapted for the assessment of observational, cohort studies (Modesti et al. [23]). [file 12966_2020_1073_MOESM3_ESM.docx]

**Additional File 3:** Risk of bias assessment using the Newcastle Ottawa scale (NOS) for cohort studies included in home environment systematic review (Adapted for the assessment of observational, cohort studies (Modesti et al 2016).

|  | Selection | | | |  | Comparability | |  | Outcome | | Total Score |
| --- | --- | --- | --- | --- | --- | --- | --- | --- | --- | --- | --- |
|  | Representativeness of the sample | Sample size | Non-respondents | Ascertainment of exposure |  | Controls for most important confounder | Controls for additional confounders |  | Assessment of outcome | Statistical test |  |
| Adachi-Mejia et al. 2007 | A* | A* | C | B* |  | A* | A* |  | D | A* | 6 |
| Anderson & Whitaker, 2010 | A* | A* | C | B* |  | A* | A* |  | A** | A* | 8 |
| Atkin et al. 2013 | A* | B | B | A** |  | A* | A* |  | A** | A* | 8 |
| Borghese et al. 2015 | B* | A* | B | A**/B*^1^ |  | A* | A* |  | A** | A* | 9/8 |
| Cameron et al. 2013 | B* | A* | B | A** |  | A* | A* |  | A** | A* | 9 |
| Cassimos et al. 2011 | C | B | C | B* |  | B | B |  | A** | B | 3 |
| Chahal et al. 2013 | B* | B | B | A** |  | A* | A* |  | A** | A* | 8 |
| Chaput et al., 2014 | B* | A* | B | A** |  | A* | A* |  | A** | A* | 9 |
| Chen et al. 2018 | B* | B | C | A** |  | A* | A* |  | A** | A* | 8 |
| Chivers et al. 2012 | C | A* | B | B* |  | B | B |  | A** | A* | 5 |
| Couch et al. 2014 | B* | B | B | A** |  | A* | A* |  | A** | A* | 8 |
| Crawford et al. 2012 | B* | B | B | B* |  | A* | A* |  | A** | A* | 7 |
| Downs et al. 2009 | B* | B | B | B* |  | B | B |  | A** | B^2^ | 4 |
| Dube et al. 2017 | A* | B | B | B* |  | A* | A* |  | A** | A* | 7 |
| Farajian, P., et al. 2014 | A* | A* | B | A**/B*^1^ |  | A* | A* |  | A** | A* | 9/8 |
| Ferrari et al. 2015 | B* | A* | B | B* |  | A* | A* |  | A** | A* | 8 |
| Ferrari et al. 2017 | B* | A* | B | A** |  | A* | A* |  | A** | A* | 9 |
| Gable and Lutz, 2000 | C | B | A* | B* |  | B | B |  | A** | A* | 5 |
| Gomes, T.N., et al. 2015 | B* | A* | B | B* |  | A* | A* |  | A** | A* | 8 |
| Gubbels et al 2011 | B* | B | A* | B* |  | A* | A* |  | A**/D^3^ | A* | 8/6 |
| Hales, D., et al. 2013 | B* | B | A* | B* |  | B | B |  | A** | A* | 6 |
| Hardy, L.L., et al. 2012 | A* | A* | B | A** |  | A* | A* |  | A** | A* | 9 |
| Heilmann, A., et al. 2017 | A* | B | B | B* |  | A* | A* |  | A** | A* | 7 |
| Humenikova & Gates, 2008 | B* | B | B | B* |  | A* | A* |  | A** | A* | 7 |
| Huynh, D.T., et al. 2011 | A* | A* | A* | B* |  | A* | A* |  | A** | A* | 9 |
| Ihmels et al. 2009 | B* | B | B | B* |  | B | B |  | A** | A* | 5 |
| Jones et al. 2009 | B* | B | B | A** |  | B | B |  | A** | A* | 6 |
| Keihner et al. 2009 | A* | B | B | B* |  | A* | A* |  | D | A* | 5 |
| Kim et al. 2014 | B* | A* | A* | B* |  | B | B |  | A** | B | 6 |
| Lane et al. 2014 | A* | A***^5^** | B | B* |  | A* | A* |  | A** | A* | 8 |
| Lehto, R., et al. 2011 | B* | B | B | B* |  | A* | A* |  | A** | A* | 7 |
| Li et al. 2014 | B* | B | B | A**/B* |  | A* | A* |  | A** | A* | 8/7 |
| Lin et al. 2019 | C | B | B | B* |  | A* | B |  | D | A* | 3 |
| Liszewska et al. 2018 | D | B | A* | A** |  | A* | A* |  | A** | A* | 8 |
| Lopez-Barron et al. 2015 | B* | B | B | B* |  | B | B |  | A** | A* | 5 |
| MacFarlane et al. 2009 | A* | B | A* | B* |  | A* | A* |  | A** | A* | 8 |
| Mathialagan et al. 2018 | B* | A* | B | B* |  | B | B |  | A** | A* | 6 |
| Mihrshahi et al. 2017 | A* | A* | B | B* |  | A* | A* |  | A** | A* | 8 |
| Moreno et al. 2011 | B* | B | B | B* |  | B | B |  | A** | A* | 5 |
| Paduano et al. 2020 | B* | B | B | B* |  | A* | B |  | D | A* | 4 |
| Palfreyman et al. 2014 | B* | B | B | B* |  | B | B |  | D | B^2^ | 2 |
| Park et al. 2019 | C | B | C | B* |  | B | B |  | A** | A* | 4 |
| Quah et al. 2018 | B* | B | A* | B* |  | A* | A* |  | A** | A* | 8 |
| Rodenburg et al. 2013 | A* | B | A* | A** |  | A* | A* |  | A** | A* | 9 |
| Rosenberg et al. 2010 | B* | B | C | B* |  | A* | A* |  | D | A* | 5 |
| Rutherford et al. 2015 | A* | A* | B | B* |  | A* | A* |  | A** | A* | 8 |
| Schalkwijk et al. 2018 | B* | B | B | B* |  | B | B |  | A** | A* | 5 |
| Schrempft, S., et al. 2015 | B* | B | A* | A** |  | A* | A* |  | D | A* | 7 |
| Serene et al. 2011 | A* | A* | B | A**/B*^1^ |  | B | B |  | A** | A* | 7/6 |
| Serrano et al. 2014 | B* | A* | B | A** |  | B | B |  | A** | A* | 7 |
| Sijtsma et al. 2015 | A* | A* | B | B* |  | A* | A* |  | A** | A* | 8 |
| Sijtsma et al. 2015 | A* | A* | A* | A** |  | B | B |  | A** | A* | 8 |
| Sirikulchayanonta et al. 2011 | B* | A* | C | B* |  | A* | A* |  | A** | A* | 8 |
| Sleddens et al. 2017 | B* | B | A* | A**/B*^1^ |  | A* | A* |  | D | A* | 7/6 |
| Taylor et al. 2011 | B* | B | B | A** |  | A* | A* |  | A** | A* | 8 |
| Terry, K. and Beck, S. 1985 | C | B | C | B* |  | B | B |  | A** | A* | 4 |
| Tiberio, S.S., et al. 2014 | B* | B | A* | B* |  | A* | A* |  | A**/D^4^ | A* | 8 |
| Torres, R., et al. 2014 | B* | A* | B | B* |  | B | B |  | A** | A* | 6 |
| Umstattd Meyer et al. 2013 | B* | B | C | B* |  | B | B |  | D | A* | 3 |
| Van Lippevelde et al. 2013 | A* | A* | B | A** |  | A* | A* |  | A** | A* | 9 |
| Vaughn, A.E., et al. 2017 | C | A* | B | A** |  | B | B |  | A** | B^2^ | 5 |
| Vaughn et al. 2019 | C | B | B | A** |  | B | B |  | A** | A* | 5 |
| ^1^Used a combination of validated and non-validated tools (adapted from existing measures) for measurement of the exposure.  ^2^Did not present data for weight, only stated that there was no significant association with weight.  ^3^At time 1 weight was measured via standardized measure and at time 2 weight was parent-reported.  ^4^A combination of standardized measurement by researcher and rest parent-reported  ^5^Available elsewhere <https://www.growingup.ie/pubs/BKMNEXT255.pdf> | | | | | | | | | | | |

**Key to star allocation** (max 10 stars)

**Selection** (max 5 stars**)**

1) Representativeness of the exposed cohort

A*= Truly representative of the general population

B* = Somewhat representative of the general population

C = Selected group of users e.g. nurses, volunteers

D = No description of sampling strategy

2) Sample size

A* = Justified and satisfactory

B = Not justified

3) Non-respondents:

A* = Comparability between respondents and non-respondents characteristics is established, and the response rate is satisfactory

B = The response rate is unsatisfactory, or the comparability between respondents and non-respondents is unsatisfactory.

C = No description of the response rate or the characteristics of the responders and the non-responders

4) Ascertainment of exposure (physical or social aspect of home environment)

A** = Self or parent-administered questionnaire (with extra validation/ reliability information reported for specific target sample)

B* = Parent or self-reported questionnaire

C = No description of the measurement tool

**Comparability** (max 2 stars**)**

The subjects in different outcome groups are comparable, based on the study design or analysis.

1) The most important confounding factor is controlled (age, sex, an indicator of socioeconomic status, energy balance behaviour, parental adiposity)^1^

A* = Yes

B = No

2) The study control for any additional confounding factors.
A* = Yes

B = No

**Outcome** (max 3 stars**)**

1) Assessment of outcome (adiposity)

A** = Clinical assessment

B** = Record linkage

C = Self-report

D = Parent-report

2) Statistical test:

A* = The statistical test used to analyze the data is clearly described and appropriate, and the measurement of the association is presented, including confidence intervals and the probability level (p value)

B= The statistical test is not appropriate, not described or incomplete.

 http://www.ncbi.nlm.nih.gov/pubmedhealth/PMH0049229

**Justification of NOS modifications**

This scale has been adapted from the Newcastle-Ottawa Quality Assessment Scale for cohort studies to perform a quality assessment of cohort studies for the systematic review, “The relationship between the home environment and child adiposity: A systematic review”.

^1^We have not selected one factor that is the most important for comparability, because the variables are not the same in each study. Thus, the principal factor should be identified for each study. Where relevant, this factor could be age, sex, an indicator of socioeconomic status, an energy balance behaviour or parental adiposity.
