# Supplementary material for: The relationship between the home environment and child adiposity: a systematic review
Source: Int J Behav Nutr Phys Act. 2021 Jan 6;18:4. doi: 10.1186/s12966-020-01073-9 (PMC7788808; doi:10.1186/s12966-020-01073-9)
Supplement: Supplementary file 4 — Additional file 4:. Cross-sectional association between physical and social aspects in the home media domain and child adiposity outcomes. [file 12966_2020_1073_MOESM4_ESM.docx]

**Additional file 4:** Cross-sectional association between physical and social aspects in the home media domain and child adiposity outcomes.

| **Author, year** | **Country** | **Age** | **Greater availability of & access to electronic devices** | **Caregiver rules & limit setting around media** | **Caregiver modelling of media use** |
| --- | --- | --- | --- | --- | --- |
| Adachi-Mejia et al. 2007 (16) | USA | 9-12 y |  |  |  |
| Atkin et al. 2013 (39) | UK | 9-11 y |  |  |  |
| Borghese et al. 2015 (40) | USA, Canada | 10 y | ***** |  |  |
| Cameron et al. 2013 (41) | 7 European countries | 10-12 y |  |  |  |
| Chahal et al. 2013. (42) | Canada | 10-11 y |  |  |  |
| Chaput et al. 2014 (43) | Canada | 9-11 y |  |  |  |
| Dube et al. 2017 (44) | Canada | 10-11 y |  |  |  |
| Farajian et al. 2014 (45) | Greece | 10-12 y |  |  |  |
| Ferrari et al. 2015 (46) | Brazil | 9-11 y |  |  |  |
| Ferrari et al. 2017 (47) | Brazil | 9-11 y |  |  |  |
| Heilmann et al. 2017 (48) | UK | 7-11 y |  |  |  |
| Gomes et al. 2015 (49) | Portugal | 9-11 y |  |  |  |
| Lane et al. 2014 (50) | Ireland | 9 y |  |  |  |
| Li et al. 2014 (51) | China | 8-10 y |  |  |  |
| Lehto et al. 2011 (52) | Finland | 9-11 y |  |  |  |
| Sijtsma et al. 2015 (23) | Netherlands | 3-4 y |  |  |  |
| Paduano et al 2020 (52) | Italy | 6-7 y |  |  |  |
| Park et al 2019 (63) | USA | 2-5 y |  |  |  |
| Anderson et al., 2010 (22) | USA | 4 y |  |  |  |
| Tiberio et al. 2014 (53) | USA | 5-9 y |  |  |  |
| Hardy et al. 2012 (27) | Australia | 5-12 y |  |  |  |
| Rutherford et al. 2015 (28) | Australia | 4-9 y |  |  |  |
| Hales et al. 2013 (33) | USA | 3–12 y |  |  |  |
| Lin et al 2019 (67) | Taiwan | 7-12 y |  |  |  |
| Vaughn et al 2019 (85) | USA | 3-12 y |  |  |  |
| Jones, et al. 2009 (34) | Australia | 2-6 y |  |  |  |
| Sleddens et al. 2017 (15) | Netherlands | 5-7 y |  |  |  |
| Taylor et al. 2011 (65) | Australia | 7-12 y |  |  |  |
| Mathialagan et al. 2018 (66) | Malaysia | 10-12 y |  |  |  |
| Rosenberg et al. 2010 (67) | USA | 5-11 y |  |  |  |
| Mihrshahi et al. 2017 (68) | Australia | 6-10 y |  |  |  |
| Keihner et al. 2009 (69) | USA | 9-11 y |  |  |  |
| Huynh, et al. 2011 (26) | Vietnam | 4-5 y |  |  |  |
| Crawford et al. 2012 (36) | Australia | 5-12 y |  |  |  |
| Ihmels et al. 2009 (35) | USA | 6-7 y |  |  |  |
| Schrempft et al. 2015 (30) | UK | 4 y |  |  |  |
| Kim et al. 2014 (24) | South Korea | 2-5 y |  |  |  |
| Gubbels et al 2011 (75) | Netherlands | 5- 7 y |  |  |  |
| **Key:** Green = negative association (lower adiposity); Red = positive association (higher adiposity); Light grey = null; White = Not measured/no data.  *****This study examined two samples, US sample and Canadian sample, differences were observed in the results. In the American sample TV in bedroom only associated with higher BF% in boys. In Canadian sample TV in bedroom associated with higher BF% in both boys and girls. | | | | | |
