# Supplementary material for: The relationship between the home environment and child adiposity: a systematic review
Source: Int J Behav Nutr Phys Act. 2021 Jan 6;18:4. doi: 10.1186/s12966-020-01073-9 (PMC7788808; doi:10.1186/s12966-020-01073-9)
Supplement: Supplementary file 5 — Additional file 5:. Cross-sectional association between physical and social aspects in the home food domain and child adiposity outcomes. [file 12966_2020_1073_MOESM5_ESM.docx]

**Additional File 5:** Cross-sectional association between physical and social aspects in the home food domain and child adiposity outcomes.

| **Author, year** | **Country** | **Age** | **Greater availability of & access to energy dense foods** | **More fruits and vegetables available and accessible in home** | **Caregiver modelling and/or support of eating** | **Caregiver rules/ limit setting around unhealthy eating** |
| --- | --- | --- | --- | --- | --- | --- |
| Cassimos et al. 2011 (54) | Greece | 9-12 y |  |  |  |  |
| Chen et al. 2018 (30) | China | 3-6 y |  |  |  |  |
| Couch et al. 2014 (13) | USA | 6-11 y |  |  |  |  |
| Downs et al. 2009 (55) | Canada | 9-12 y |  |  |  |  |
| Humenikova 2008 (56) | Czech. USA | 10-11 y |  |  |  |  |
| Gable 2000 (9) | USA | 6-10 y |  |  |  |  |
| Lopez-Barron et al. 2015 (57) | Mexico | 10-11 y | **^*^** | ^*^ |  |  |
| MacFarlane et al. 2009 (38) | Australia | 5-6 & 10-12 y |  |  |  |  |
| Terry 1985 (58) | USA | 8-12 y |  |  |  |  |
| Palfreyman et al. 2014 (59) | UK | 1-8 y |  |  |  |  |
| Van Lippevelde et al. 2013 (60) | 7 European Countries | 10-12-y |  |  |  |  |
| Vaughn et al. 2017 (31) | USA | 3-12 y |  |  |  |  |
| Mihrshahi et al. 2017 (68) | Australia | 6-10 y |  |  |  |  |
| Keihner et al. 2009 (69) | USA | 9-11 y |  |  |  |  |
| Huynh et al. 2011 (26) | Vietnam | 4-5 y |  |  |  |  |
| Serene et al. 2011 (70) | Kuala Lumpur | 9-12 y |  |  |  |  |
| Serrano et al. 2014 (71) | Puerto Rico | 12 y |  |  |  |  |
| Torres et al. 2014 (74) | Puerto Rico | 12 y |  |  |  |  |
| Ihmels et al. 2009 (35) | USA | 6-7 y |  |  |  |  |
| Quah et al 2018 (80) | Singapore | 5 y |  |  |  |  |
| Kim et al. 2014 (24) | South Korea | 2-5 y |  |  |  |  |
| Gubbels et al 2011 (29) | Netherlands | 5- 7 y |  |  |  |  |
| **Key:** Green = negative association (lower adiposity); Red = positive association (higher adiposity); Light grey = null; White = Not measured/no data.  * OW/OB associated with lower availability of Energy Dense Foods (EDF) at home (OR 0.56, p<.001). OW/OB associated with increased odds of availability of fruits and vegetables (OR = 1.10, p = 0.035). | | | | | | |
