# Supplementary material for: The relationship between the home environment and child adiposity: a systematic review
Source: Int J Behav Nutr Phys Act. 2021 Jan 6;18:4. doi: 10.1186/s12966-020-01073-9 (PMC7788808; doi:10.1186/s12966-020-01073-9)
Supplement: Supplementary file 6 — Additional file 6:. Cross-sectional association between physical and social aspects in the home PA domain and child adiposity outcomes. [file 12966_2020_1073_MOESM6_ESM.docx]

**Additional File 6:** Cross-sectional association between physical and social aspects in the home PA domain and child adiposity outcomes.

| **Author, year** | **Country** | **Age** | **Greater access to and availability of PA** | **Caregiver modelling & support of PA** |
| --- | --- | --- | --- | --- |
| Chivers et al., 2012 (83) | Australia | 1-10 y |  |  |
| Sijtsma et al. 2015 (85) | Netherlands | 3-4 y |  |  |
|  |  |  |  | ***^1^** |
| Liszewska et al. 2018 (84) | Poland | 6-11 y |  |  |
| Schalkwijk et al. 2018 (37) | UK | 3-7 y |  |  |
| Umstattd Meyer et al. 2013 (82) | US/Mexico border | 6-11 y |  |  |
| Hales et al. 2013 (38) | USA | 3–12 y |  |  |
| Jones, et al. 2009 (39) | Australia | 2-6 y |  |  |
| Sleddens et al. 2017 (15) | Netherlands | 5-7 y |  |  |
| Taylor et al. 2011 (66) | Australia | 7-12 y |  |  |
| Mathialagan et al. 2018 (64) | Malaysia | 10-12 y |  |  |
| Rosenberg et al. 2010 (51) | USA | 5-11 y |  |  |
| Serene et al. 2011 (78) | Kuala Lumpur | 9-12 y |  |  |
| Serrano et al. 2014 (79) | Puerto Rico | 12 y |  |  |
| Torres et al. 2014 (73) | Puerto Rico | 12 y |  |  |
| Crawford et al. 2012 (47) | Australia | 5-12 y |  |  |
| Ihmels et al. 2009 (40) | USA | 6-7 y |  |  |
| Kim et al. 2014 (29) | South Korea | 2-5 y |  |  |
| Gubbels et al 2011 (34) | Netherlands | 5- 7 y |  |  |
| Vaughn et al 2019 (68) | USA | 3-12 y |  | ***^2^** |
|  |  |  |  |  |
| **Key:** Green = Negative association (lower adiposity); Red = Positive association (higher adiposity); Light grey = null; White = Not measured/no data.  *^1^Sijtsma et al. (2015) examined caregiver modelling of PA; differences were observed between the types of activities modelling. No association between caregiver modelling of PA and BMI z-score or waist circumference. Caregiver modelling of commuting to school/work by bike or walking associated with lower BMI Z-score (r = −0.062).  *^2^ Vaughn et al. (2019) examined caregiver encouragement, support, and modelling of PA as separate constructs. Caregiver encouragement and support of PA were associated with lower BMI percentile. Caregiver modelling of PA was not associated with child adiposity outcome. | | | | |
